# Supplementary material for: Arithmetic optimization based secure intelligent clustering algorithm for Vehicular Adhoc Network
Source: PLoS One. 2024 Sep 12;19(9):e0309920. doi: 10.1371/journal.pone.0309920 (PMC11392382; doi:10.1371/journal.pone.0309920)
Supplement: Attachment — Submitted filename: Response to Reviewers Comments- AOA.docx [file pone.0309920.s002.docx]

**Authors’ response to the reviewers’ comments**

**Title: Arithmetic Optimization based Secure Intelligent Clustering Algorithm for Vehicular Adhoc Network**

**Journal: PLOS ONE**

The authors are thankful to the reviewers and the associate editor for providing their useful and constructive comments. We have taken carefully your valuable and prolific suggestions into consideration while preparing our revision. The modified paper got much improvement and clarity by incorporating the comments. We hope that the enclosed revised paper will meet the requirements suggested by the reviewers and the associate editor.

**Note:** There are two versions of the manuscript i.e. **manuscript- (clean copy)** and **manuscript- (track changes)**.

**Comments from the editors and reviewers:**

-**Reviewer:1**

 Comments:

**Comment 1**: The article lacks proper organization with the absence of headings and subheadings throughout its content.
Abstract:
i. Clarify the specific meta-heuristic algorithm employed by AOACNET.
ii. Provide more details on the threshold condition for authenticating nodes during cluster formation.
iii. Specify the nature of the extensive simulation results and the metrics used for evaluation.

**Answer:**

We are thankful to the respectable reviewer for his concern for improvement in the manuscript. The format, language and organization of the paper is improved as per the direction of the respectable reviewer.

Moreover, in the revised manuscript, we clarify the use of specific meta-heuristic algorithm for the proposed clustering algorithm. We also provide a detail on threshold value which is set to 0. If a vehicle performance value is greater than the threshold value, the vehicle will consider an authentic vehicle and will be able to be a part of the cluster. In addition, we also specify the evaluation metric and simulation parameters for extensive simulation.

**Comment 2:** Introduction	:
i. Elaborate on the unique challenges posed by the dynamic network topology in VANETs.
ii. Include a concise comparison with existing clustering methods in VANETs to emphasize the innovation of AOACNET.
iii. The section provides a comprehensive overview of the challenges in Vehicular Adhoc Networks (VANET) and the importance of secure vehicular clustering. However, consider breaking down the lengthy paragraphs into smaller ones for improved readability

**Answer:** We are thankful to the respectable reviewer for his concern for improvement in the manuscript. The introduction section is revised as per the direction of the respectable reviewer.

**Comment 3**: Motivation and Problem Statement:
The motivation for secure vehicular clustering is well-stated, particularly emphasizing the impact of malicious nodes on passenger safety. Consider providing specific examples or scenarios to illustrate the severity of the problem.

**Answer:**

We are thankful to the respectable reviewer for his positive comments and his concern for improvement in the manuscript. The scenarios are provided as per the direction of respectable reviewer.

**Comment** 4: Introduction to Vehicular Clustering:
The introduction to vehicular clustering is informative. However, you may want to elaborate more on the benefits and challenges of clustering in VANETs. How does clustering address the issues of dynamic network topology and fast vehicle movement?

**Answer:**

We are thankful to the respectable reviewer for his positive comments and his concern for improvement in the manuscript. In the revised manuscript, we discuss the benefits and challenges of vehicular clustering. In addition, we also discuss how clustering address the issues of dynamic network topology.

**Comment 5**: Importance of Optimal Clustering:
The importance of optimal clustering is highlighted, touching on factors like communication efficiency and resource utilization. Consider providing more concrete examples or studies that demonstrate the impact of optimal clustering on VANET performance.

**Answer:**

We are thankful to the respectable reviewer for his concern for improvement in the manuscript. In the revised manuscript we provide more details regarding the impact of optimal clustering on VANET performance.

**Comment 6**:   Introduction to AOA (Arithmetic Optimization Algorithm)
The introduction to AOA is clear, but you might want to briefly explain why AOA is chosen over other meta-heuristic algorithms. What specific characteristics of AOA make it suitable for vehicular clustering?

**Answer:**

We are thankful to the respectable reviewer for his concern for improvement in the manuscript. In the revised manuscript, we briefly add the characteristics of AOA that make it effective method for vehicular clustering.

**Comment 7**:   Clarity and Organization:

The literature review provides a comprehensive overview of clustering protocols in vehicular networks. However, consider breaking down the section into smaller subsections for better organization, making it easier for readers to navigate through the content.

**Answer:**

We are thankful to the respectable reviewer for his positive comment and his concern for improvement in the manuscript. Basically the literature review is already divided into section and subsection for better organization and clarity. Broadly it is divided into three section i.e.. General purpose clustering, domain-specific clustering algorithm and meta-heuristic algorithm. The domain specific clustering algorithm is further divided into four subsection i.e. Cluster-based Routing algorithm, MAC-based clustering algorithm, secure clustering algorithm and QoS-aware clustering algorithm. Each of these subsection includes various high qualities papers.

**Comment 8**:   Taxonomy of clustering protocol

The classification of clustering protocols into general-purpose and domain-specific categories is clear. Consider providing a brief summary or transition statement before introducing Fig 3 to prepare the reader for the taxonomy.

**Answer:**

We are thankful to the respectable reviewer for his positive comment and his concern for improvement in the manuscript. The taxonomy of clustering protocol shows the division of clustering algorithm into three division i.e., general-based, domain specific and meta-heuristic and their subdivision that are already briefly discuss just before the inclusion of figure 3. However, in the revised manuscript we add a brief statement about meta-heuristic clustering algorithm.

**Comment 9**:  Fig 3- Classification of clustering protocol

The taxonomy figure is a valuable addition. However, ensure that the figure is adequately explained in the text. Each category and its significance should be briefly discussed.

**Answer:**

We are thankful to the respectable reviewer for his positive comment and his concern for improvement in the manuscript. All the categories and sub categories of Fig 3 are significantly explained in the manuscript.

**Comment 10**: General Purpose Clustering Protocol:

The descriptions of general-purpose clustering algorithms are informative. Consider adding a sentence or two after each algorithm to highlight its strengths or limitations in the context of vehicular networks.

**Answer:**

We are thankful to the respectable reviewer for his positive comment and his concern for improvement in the manuscript. In the revised manuscript we highlight the strengths and weakness of each general purpose clustering protocol as suggested by the respectable reviewer.

**Comment 11**: Domain specific Clustering Protocol:

The categorization of domain-specific clustering protocols into routing, security, MAC, and QoS is effective. Ensure that each sub-section provides a concise yet comprehensive overview of the protocols within that domain.

**Answer:**

We are thankful to the respectable reviewer for his positive comment and his concern for improvement in the manuscript. In the revised manuscript, we provide the concise and comprehensive detail of each sub-category as per the direction of the respectable reviewer.

**Comment 12**: Routing Protocol

The description of clustering in the context of vehicular routing is clear. Consider adding a brief sentence about the importance of clustering in improving routing efficiency in VANETs.

**Answer:**

We are thankful to the respectable reviewer for his positive comment and his concern for improvement in the manuscript. In the revised manuscript, we briefly discuss the importance of clustering in improving routing efficiency in VANET as per the direction of the respectable reviewer.

**Comment 13**: Security Protocol

The overview of security protocols is well-structured. However, for each protocol, consider providing a sentence about how clustering enhances security in vehicular networks.

**Answer:**

We are thankful to the respectable reviewer for his positive comment and his concern for improvement in the manuscript. This section is revised as per the instruction of the respectable reviewer.

**Comment 14**: Cluster-based MAC Protocol

The section on MAC protocols is comprehensive. Consider briefly explaining how cluster-based MAC protocols address challenges like fair channel access in vehicular networks.

**Answer:**

We are thankful to the respectable reviewer for his positive comment and his concern for improvement in the manuscript. In the revised manuscript, we explain the procedure of fair channel access using cluster-based MAC protocol.

**Comment 15**: QoS assurance Protocol

The discussion on QoS assurance protocols is well-done. Consider emphasizing the role of clustering in maintaining QoS metrics and how it contributes to the overall network performance.

**Answer:**

We are thankful to the respectable reviewer for his positive comment and his concern for improvement in the manuscript. In the revised manuscript, we explain the role of clustering in maintaining the QoS of vehicular network as per the instruction of the respectable reviewer.

**Comment 16**: Meta-heuristic based clustering algorithm

The explanation of meta-heuristic algorithms is clear. Consider adding a sentence after each algorithm to highlight its specific advantages in the context of clustering for vehicular networks.

**Answer:**

We are thankful to the respectable reviewer for his positive comment and his concern for improvement in the manuscript. In the revised manuscript, we highlight the specific advantage of each meta-heuristic based clustering algorithm as per direction of the respectable reviewer.

**Comment 17**: Arithmetic Optimization Algorithm

The introduction of AOA is well-described. Consider adding a sentence or two about why AOA is chosen as the focus of the paper and how it differs from other meta-heuristic algorithms.

**Answer:**

We are thankful to the respectable reviewer for his positive comment and his concern for improvement in the manuscript. In the introduction section, we discuss the same points in details that briefly explain the reasons behind selection of AOA over other meta-heuristic algorithms. In this section, we only describe the working of AOA algorithm.

**Comment 18**: Distribution behavior of AOA

The explanation of the distribution behavior of AOA is clear. Consider providing a brief example or analogy to help readers conceptualize the diversification and intensification phases.

**Answer:**

We are thankful to the respectable reviewer for his positive comment and his concern for improvement in the manuscript. In the revised manuscript, we explain the role of clustering in maintaining the QoS of vehicular network as per the instruction of the respectable reviewer.

**Comment 19**: Explanation of MOA Function:

The MOA function is introduced, but there's no detailed explanation of its purpose or significance. Provide more context on how it contributes to the search space creation.

**Answer:**

We are thankful to the respectable reviewer for his concern for improvement in the manuscript. Math Optimizer function is a linear function whose value decides the usage of two important features of AOA algorithm i.e., exploration and exploitation phase. The value of the MOA is updated using eq 1 of paper in each iteration from 0.2 to 0.9. The purpose of the MOA is to give an equal chance to the algorithm to choose between exploration or exploitation search phase. In case of rand > MOA, the algorithm uses exploration phase to explore the search space for diverse potentials solutions and in other case, algorithm employs exploitation phase to conduct the search for optimal solution within the search space.

**Comment 20**: Explanation of Search Space

Clarify how the search space is represented and how candidate solutions move within this space. A visual representation or example would be beneficial.

**Answer:**

We are thankful to the respectable reviewer for his kind suggestion and his concern for improvement in the manuscript. The search space is created with a set of candidate solution. Each point in the search space represent a candidate solution which is encapsulated with the cluster matrix. Each candidate solution has a different set of vehicular clusters which is evaluated using the fitness function. In each iteration, the objective function determines the optimal solution. Afterward the candidate solution moves around the best optimal solution using exploration or exploitation search phase.

**Comment 21**: Explanation of secure cluster formation (Lines 416-443):

The section is detailed, but consider breaking down complex processes, such as Roulette Wheel Selection (RWS), into smaller steps for better understanding.

**Answer:**

We are thankful to the respectable reviewer for his concern for improvement in the manuscript. The subsection is revised as per the direction of the respectable reviewer.

**Comment 22**: Roulette Wheel Selection

Provide a bit more context or a simple example of how RWS works in the context of secure cluster formation.

**Answer:**

We are thankful to the respectable reviewer for his concern for improvement in the manuscript. We provide more details on RWS in the context of secure cluster formation.

**Comment 23**: Explanation of objective function

Offer a brief explanation of why a multi-objective fitness function is chosen and how ψ and μ contribute to the evaluation of solutions.

**Answer:**

We are thankful to the respectable reviewer for his concern for improvement in the manuscript. The multi-objective function is chosen, the vehicular clustering is considered as multi-objective problem, where multiple objectives need to be maximized or minimized simultaneously. In this problem, we use two objectives, one is distance neighbor and the other is delta difference. Both objectives are necessary for vehicular cluster optimization, and for communication efficiency.

**Comment 24**: Delta Difference (Equation 7)
Provide a bit more explanation or intuition behind the delta difference concept.

**Answer:**

We are thankful to the respectable reviewer for his concern for improvement in the manuscript. In the revised manuscript, we provide the details of delta difference. Basically, the value for the delta varies subject to the network condition i.e., for a highly dense network, the delta value will be high and vice versa. The minimum value of delta difference is required to achieve optimization, scalability, and reliability of the communication protocol. Each cluster tries to cover maximum number of cluster members but below delta value in an attempt to optimize the number of clusters.

**Comment 25**: Clarification on Exploration and Exploitation:
Clearly explain the roles of exploration and exploitation in the context of repositioning. It might help to provide an example or visual aid.

**Answer:**

We are thankful to the respectable reviewer for his concern for improvement in the manuscript. Exploration and exploitation phases are the two key features of meta-heuristic algorithms. Exploration, randomly explore the regions of the search space for wide range of potential solutions. In the exploration search phase, the algorithm randomly move within the search space to discover the regions that may contain potential solution. Exploitation, intensify the regions found by the exploration search phase. In the exploitation phase, the algorithm move to regions found in the previous phase, to fine-tune the solution.

**Comment 26**: Mathematical Notation
Ensure that all symbols used in equations are defined or referenced in Table 1 for clarity.

**Answer:**

We are thankful to the respectable reviewer for his concern for improvement in the manuscript. All the symbols are referenced and defined clearly.

**Comment 27**: Explanation of Exploration and Exploitation:
Offer more insight into why certain operators are considered explorative or exploitative and how they affect the search process.

**Answer:**

We are thankful to the respectable reviewer for his concern for improvement in the manuscript. In arithmetic operation, certain operators like multiplication and division introduces large changes to the variable’s values. These operators have high dispersion and distributed values that restrict the operators from approaching the target. Hence, the division and multiplication operators are considered exploration operators. The operators like addition and subtraction introduce less changes to the value of variable. We cannot conclude the expression evaluation without final arithmetic operation, means these operators are mainly used to fine-tune the solution that are already close to the optimal region. Therefore addition and subtraction operators are considered exploitative operator.
The search process is mainly effected by the unbalance phases of exploration and exploitation means that there is no balancing between the exploration and exploitation search strategies.

**Comment 28**: Stochastic Scaling Coefficients:
Provide a brief explanation of why stochastic scaling coefficients are necessary and how they impact the algorithm.

**Answer:**

We are thankful to the respectable reviewer for his concern for improvement in the manuscript. Stochastic scaling co-efficient promotes diversity in the candidate solution by introducing randomness in the scaling process. Sometime, same type of changes in the candidate solution, may cause the algorithm to converge earlier. Therefore stochastic co-efficient is used to avoid premature convergence. Moreover, the co-efficient also help the algorithm to maintain a balance between exploration and exploitation search phase.

**Comment 29:** Pseudo Code of AOACNET, Initialization (Lines 1-3):
Clarify the purpose of parameters like 'a' and 'ϱ' in the initialization step.
**Answer:** We are thankful to the respectable reviewer for his concern for improvement in the manuscript. 'a' is a sensitive parameter used to escalate the exploitative accuracy of the searching process, and ϱ is a control parameter used to adjust the search strategy in the search space to avoid local stagnation. These are the parameters that need to be initialized in the initialization phase.

**Comment 30:** Neighbor Matrix Calculation (Line 4):
Provide more details on how the Euclidean distance is used to compute the neighbor matrix. Since there are many distance calculation methods are available example Manhattan Distance, Minkowski Distance, and Hamming Distance.

**Answer:** We are thankful to the respectable reviewer for his kind concern for improvement in the manuscript. We have used standard Euclidean distance that computes the distance between two points in the two dimensional problem space.
**Comment 31:** Cluster Matrix Creation (Lines 10-13):
Clarify how the candidate solution contains a different number of optimal clusters.
**Answer:** We are thankful to the respectable reviewer for his kind concern for improvement in the manuscript. The candidate solution contain different set of clusters because of the stochastic behavior and different location of solution within the search space.

**Comment 32:** Distribution Behavior of Arithmetic Operators (Lines 14-16):
Offer more details on how the distribution behavior of operators is determined and its significance in finding near-optimal locations.

**Answer:** We are thankful to the respectable reviewer for his kind concern for improvement in the manuscript. In arithmetic operation, arithmetic operators are distributed based on the dispersion and ability to change the value of variable. Some operators (Multiplication and division) has high dispersion and produces high changes to the value of the variable. These operators perform the role of exploration phase in optimization, other operators (addition, subtraction) has low dispersion and produces less changes to the value of the variable. These operator conclude the evaluation of the expression. That is why, these operators performed the role of exploitation search phase in optimization. Together operation of these operators, enable the algorithm to find the optimal solution. (These points are explained in exploitation and exploration search phases).

**Comment 33:** Specify what the final output represents—is it the best solution in terms of the fitness function?

**Answer:** We are thankful to the respectable reviewer for his kind concern for improvement in the manuscript. The final output represent the best solution in terms of optimal number of secure vehicular clusters.

**Comment 34:** Consider adding comments within the pseudo-code for better readability.
**Answer:** We are thankful to the respectable reviewer for his kind suggestion for improvement in the manuscript. The comments are added within the pseudo code as per the direction of the respectable reviewer.

**Comment 35:** Provide more contextual examples or figures where applicable to aid understanding.
**Answer:** We are thankful to the respectable reviewer for his kind suggestion for improvement in the manuscript. The figure as an example is included in the revised manuscript to aid the readers in understand the complexity of distribution behavior of arithmetic operators

**Comment 36:** Ensure that all symbols, variables, and parameters are consistently defined and explained. Review the algorithm for any potential edge cases or scenarios not covered in the explanation.
**Answer:** We are thankful to the respectable reviewer for his kind suggestion for improvement in the manuscript. We checked the manuscript thoroughly, all the symbols, parameters and variables are properly defined. Moreover, all the explanations of an algorithm are covered in the manuscript.

**Comment 37:** Consider including a summary or conclusion at the end of the section to reinforce key points.
**Answer:** We are thankful to the respectable reviewer for his kind suggestion for improvement in the manuscript. Each section is summarized as per the direction of the respectable reviewer.

**Comment 38:** MATLAB Version: Specify the MATLAB toolbox or specific functions used in the implementation. This adds transparency and helps others replicate the results.
**Answer:** We are thankful to the respectable reviewer for his kind suggestion for improvement in the manuscript. We have used different functions to implement the proposed algorithm However, most of the functions are user defined function to implement the desired functionality i.e., cluster matrix creation, objective matrix creation etc.

**Comment 39:** Experimental Setup (Lines 538-547): Hardware Specifications:
Include more details about the laptop's specifications, such as the processor model and architecture, to provide a clearer understanding of the computational resources.
**Answer:** We are thankful to the respectable reviewer for his kind concern for improvement in the manuscript. In the revised manuscript, we add more detail on system specification such as architecture, processor model and manufacturer etc.

**Comment 40:** Simulation Parameters (Table 6): Clearly explain the significance and impact of each simulation parameter on the results. This will help readers understand the experimental design better.

**Answer:**

We are thankful to the respectable reviewer for his kind concern for improvement in the manuscript. Each simulation parameter plays an important role in finding the optimal result like control parameter is used to avoid local optima, sensitive parameter that escalate the searching process, transmission range and network size etc.

**Comment 41:** AOACNET Algorithm (Lines 519-536): Initialization (Line 1): Explain the significance of the "direction and speed of automobiles" in the initialization step.
**Answer:** We are thankful to the respectable reviewer for his kind concern for improvement in the manuscript. Each vehicle is also initialized within the search space along with the speed and direction. The speed of vehicle is set of 30ms^-1^.

**Comment 41:** Cluster Formation (Lines 9-13): Provide more details on how the cluster matrix is initialized and how the cluster formation process contributes to the overall algorithm.

**Answer:** We are thankful to the respectable reviewer for his kind concern for improvement in the manuscript. Cluster formation is the second phase of the algorithm. Cluster formation means creation of a cluster matrix which is created based on the performance value of each vehicle. The same point is addressed in the cluster formation section. In the beginning, cluster matrix is empty means there is no clusters within the candidate solution. The performance of the algorithm mainly depends upon the cluster formation phase. For instance, if a cluster contain malicious node, then it can disturb the performance of the network application.

**Comment 42:** Update Positions (Lines 13-15): Clarify how the positions are updated, and explain the role of the MOA and MOP functions in this context.

**Answer:** We are thankful to the respectable reviewer for his kind concern for improvement in the manuscript. In each iteration, the position of candidate solutions are updated towards the best candidate solution (best so far) using exploration update strategy and exploitation update strategy. MOA is math optimizer accelerated function which is used to give a direction to the algorithm to select either exploration search strategy or exploitation search strategy. MOP is a math optimizer probability which is used to escalate the accuracy of each search strategy. (Position updating mechanism are discussed in detail in exploitation search strategy and exploration search strategy)

**Comment 43:** Results Discussion (Lines 548-574): Performance Metrics: Elaborate on the significance of using "number of optimal clusters" and "load balancing" as performance metrics. Why are these metrics chosen, and how do they relate to the goals of the clustering algorithm?
**Answers:** We are thankful to the respectable reviewer for his kind concern for improvement in the manuscript. Number of optimal clusters is used as performance metrics to evaluate the proposed algorithm, because number of clusters effect the reliability of vehicular application i.e., too many clusters may result in unwanted end-to-end delays, and produces high overhead on the network due to formation and maintenance of clusters. While an optimal number of clusters may result in intermittent connectivity and reduces routing cost and overhead. As the objective of the proposed algorithm is to find optimal clusters. Therefore, number of optimal clusters is chosen as performance metric. We also used load balancing as performance metrics because load balancing also effect the performance of the vehicular network i.e., if a cluster is overwhelmed with lots of communication and computations task, then the algorithm increases scarce wireless resource utilization. Therefore, we have chosen load balancing as performance metric.

**Comment 44:** Figures 5 and 6: Provide axis labels and legends to enhance the readability of the figures. This will make it easier for readers to interpret the results.

**Answers:** We are thankful to the respectable reviewer for his kind concern for improvement in the manuscript. The labels and legends are provided as per the direction of the respectable reviewer.

**Comment 45:** Relation between Transmission Range and Clusters:
Clarify the observed relationship between transmission range and the number of optimal clusters. A more detailed explanation would add depth to the interpretation.

**Answers:** We are thankful to the respectable reviewer for his kind concern for improvement in the manuscript.

There is a strong direct relationship between the transmission range and the number of optimal clusters. Upon increasing the transmission range, the total set of optimal clusters drops because the coverage area of each CH increases, which covers more members in its purlieu and vice versa.

**Comment 46:** Discussion on Simulation Area (Lines 566-572): Elaborate on the relationship between the simulation area and the number of optimal clusters. Why does increasing the simulation area result in more optimal clusters?

**Answers:** We are thankful to the respectable reviewer for his kind concern for improvement in the manuscript.

There is a strong relationship between simulation area and number of optimal clusters i.e., increasing the simulation area also increases the set of optimal clusters because the vehicles are deployed far away from each other, as a results only nodes are covered within the transmission range of a CH. Hence total number of optimal clusters increase for a given vehicular network.

**Comment 47:** Numerical Analysis (Lines 575-622):
Provide a bit more context around the numerical analysis, especially the percentages mentioned in Table 7. What do these percentages represent?

**Answers:** We are thankful to the respectable reviewer for his kind concern for improvement in the manuscript.

The table has 5 columns, where columns 2 and 3 represent the percentage of clusters selected in each network area. Columns 4 and 5 represent the percentage of cluster heads and cluster members for all network areas.

**Comment 48:** Load Balance Factor (LBF) (Lines 602-616): Equation 11:
Explain the significance of ϕc and how it influences the load balance factor. A more detailed explanation of the formula would be helpful.

**Answers:** We are thankful to the respectable reviewer for his kind suggestion and his concern for improvement in the manuscript. ϕc represent the size of candidate solution means total number of optimal clusters. The size of candidate solution has direct impact on the load balance factor, if a candidate solution has large size i.e., contain large number of clusters then the load on each cluster is minimum because CH are serving only few nodes as their coordinator. Hence load is not balanced across all clusters. On other hand, if size of a candidate solution is optimal, then the load on each cluster will be balanced. As a result overall, system performance will be improved.

**Comment 49:** Conclusion (Lines 625-646): Integration of Emerging Technologies: Provide more details on how the proposed algorithm could adapt to emerging technologies such as 5G, 6G, and Flying Adhoc Networks (FANET). What specific challenges and optimizations are anticipated in these scenarios?

**Answers:** We are thankful to the respectable reviewer for his kind concern for improvement in the manuscript. The proposed clustering algorithm can adapt to emerging technologies by considering the unique challenges faced by 5G and 6G communication architectures. 5G and 6G communication architectures introduce high data rates and have low latency requirements. So adapting the proposed clustering algorithm to these emergent technologies would not only require the optimization of clusters but also require consideration of dynamic cluster reshaping based on high data rates and low latency. Flying Adhoc Networks (FANET) faced unique challenges such as high mobility and 3D spatial consideration. These challenges increase the complexity of cluster formation and cluster maintenance. So the proposed algorithm must consider the mobility of nodes to proactively adjust cluster configuration. Moreover, the adaptation of the proposed clustering algorithm to FANET would require spatial awareness into the solution to optimize the communication path in 3D space.

**Comment 50:** Overall Suggestions: Consistent Terminology:
Ensure that terminology is used consistently throughout the section. For example, use either "optimal clusters" or "optimal clusters" consistently.

**Answer:** We are thankful to the respectable reviewer for his kind suggestion and his concern for improvement in the manuscript. In the revised manuscript, we use the word optimal clusters throughput the paper as per the suggestion of the respectable reviewer.

**Comment 51:** Visual Aids: Consider adding visual aids, such as flowcharts or diagrams, to illustrate the algorithm's steps and the experimental setup.

**Answer:** We are thankful to the respectable reviewer for his kind suggestion and his concern for improvement in the manuscript. In the revised manuscript, we add a flowchart and some diagrams to aid the reader in understanding the complexity of the manuscript.

**Comment 52:** Interpretation Guidance: Provide more guidance on interpreting the figures and tables. Explain the observed trends and their implications for the algorithm's performance.

**Answer:** We are thankful to the respectable reviewer for his kind suggestion and his concern for improvement in the manuscript. In the revised manuscript, all the figures and tables are defined as per the direction of the respectable reviewer.

**Comment 53:** Statistical Analysis:
If applicable, consider including statistical analysis of results (e.g., standard deviations) to quantify the robustness and reliability of the algorithm.

**Answers:** We are thankful to the respectable reviewer for his kind concern for improvement in the manuscript. We include a numerical analysis of the proposed algorithm using a mean to quantify the reliability of the algorithm in terms of number of optimal clusters.

Future Work:
**Comment 53:** Expand on potential directions for future work, especially in terms of refining the algorithm or addressing specific challenges in vehicular networks.

**Answers:** We are thankful to the respectable reviewer for his kind concern for improvement in the manuscript. We can extend the proposed algorithm by incorporating machine learning techniques to identify traffic patterns, vehicle behavior and network condition to create efficient clusters.

Reviewer #2:

**Comment 1:** There is no security analysis available in this manuscript. The authors are instructed to analysis the paper to prepare the security analysis part.

**Answers:** We are thankful to the respectable reviewer for his kind concern for improvement in the manuscript. The security part of the proposed solution is analyzed in terms of end-to-end delay and network overhead.
