# Supplementary material for: Arithmetic optimization based secure intelligent clustering algorithm for Vehicular Adhoc Network
Source: PLoS One. 2024 Sep 12;19(9):e0309920. doi: 10.1371/journal.pone.0309920 (PMC11392382; doi:10.1371/journal.pone.0309920)
Supplement: Attachment — Submitted filename: Response to Reviewers Comments-AOA.docx [file pone.0309920.s003.docx]

**Authors’ response to the reviewers’ comments**

**Title: Arithmetic Optimization based Secure Intelligent Clustering Algorithm for Vehicular Ad-hoc Network**

**Journal: PLOS ONE**

The authors are thankful to the reviewers and the associate editor for providing their useful and constructive comments. We have taken carefully your valuable and prolific suggestions into consideration while preparing our revision. The modified paper got much improvement and clarity by incorporating the comments. We hope that the enclosed revised paper will meet the requirements suggested by the reviewers and the associate editor.

**Note:** There are two versions of the manuscript i.e. **manuscript- (clean copy)** and **manuscript- (track changes)**.

**Comments from the editors and reviewers:**

-**Reviewer:1**
**Comment 1**: **The author has diligently taken into consideration and appropriately addressed all the comments therefore, I recommend accepting the revised manuscript**.

**Answer:**

We are thankful to the respectable reviewer for considering the comments and accepting the revised manuscript.

**-Reviewer:2**

 Comments:

**Comment 1**: **No proper security analysis with respect to attack scenarios. Refer the following papers for security analysis.**

**Answer:**

We are thankful to the respectable reviewer for his concern for improvement in the manuscript. We carefully consider the comments of the respectable reviewer and analyze the security of the proposed algorithm concerning different attack scenarios i.e., resistance against impersonation attack, replay attack, Sybil node attack, and bogus message attack. The dynamic performance attribute of each vehicle and the valid timestamp and cryptographic nonce of each message enables the proposed algorithm to effectively resist security attacks.

Moreover, we also analyze the performance of the proposed algorithm in terms of computational time which refers to the time taken by the algorithm to authenticate a single vehicle. The simulation results declare that with minimal calculations, the proposed algorithm consumes less time as compared to other schemes.
