# Supplementary material for: Arithmetic optimization based secure intelligent clustering algorithm for Vehicular Adhoc Network
Source: PLoS One. 2024 Sep 12;19(9):e0309920. doi: 10.1371/journal.pone.0309920 (PMC11392382; doi:10.1371/journal.pone.0309920)
Supplement: Attachment — Submitted filename: Response to Reviewers Comments-AOA-R3.docx [file pone.0309920.s004.docx]

**Authors’ response to the reviewers’ comments**

**Title: Arithmetic Optimization based Secure Intelligent Clustering Algorithm for Vehicular Ad-hoc Network**

**Journal: PLOS ONE**

The authors are thankful to the reviewers and the associate editor for providing their useful and constructive comments. We have taken carefully your valuable and prolific suggestions into consideration while preparing our revision. The modified paper got much improvement and clarity by incorporating the comments. We hope that the enclosed revised paper will meet the requirements suggested by the reviewers and the associate editor.

**Note:** There are two versions of the manuscript i.e. **manuscript- (clean copy)** and **manuscript- (track changes)**.

**Comments from the editors and reviewers:**

-**Reviewer:1**
**Comment 1**:Please cite the following articles that are related to the proposed article: 1. Blockchain-based batch authentication protocol for Internet of Vehicles 2. Blockchain-based mutual-healing group key distribution scheme in unmanned aerial vehicles ad-hoc network.

**Answer:**

We are thankful to the respectable reviewer for his concern for improvement in the manuscript. The suggested articles are cited in the Literature review section (subsection –secure clustering algorithm) as per the suggestion of the respectable reviewer.

**-Reviewer:2**

**Comment 1**: No comments from my side. This paper can be accepted in its current form**.**

**Answer:**

We are thankful to the respectable reviewer for accepting the revised manuscript.

**Reviewer:3**
**Comment 1**: In-depth Discussion on Security Analysis: "Please further discuss the performance of the proposed algorithm under various malicious attacks in the security analysis section. For example, can the algorithm effectively respond to rapid changes in malicious nodes in highly dynamic environments? Additionally, considering the potential diversity of malicious node attack patterns, could you add an analysis of the algorithm's ability to detect other types of attacks, such as node hijacking?"

**Answer:** We are thankful to the respectable reviewer for his concern for improvement in the manuscript.

The proposed algorithm is designed to operate efficiently in a highly dynamic vehicular network. The performance attribute of each vehicle is dynamically updated based on real-time communication, and even after the selection of a cluster member, the RSU continuously monitors the behavior of cluster member. This includes, observing irregular patterns, sudden message spikes and sudden variation is performance attribute). Therefore due to real-time continuous monitoring and dynamic update mechanism of performance value, the proposed algorithm is able to respond to rapid changes in malicious nodes.

The proposed algorithm is versatile in detecting a range of malicious attacks including node hijacking attack. The security analysis of node hijacking attack is included in the manuscript as per the suggestion of the respectable reviewer.

**Comment 2**: Algorithm Efficiency Comparison: "In the comparison of computation time, it is recommended to include some of the latest commonly used security algorithms in addition to the existing comparison algorithms. This will help to more comprehensively demonstrate the efficiency advantages of the proposed algorithm."

**Answer:** We are thankful to the respectable reviewer for his kind suggestion. The set of benchmark algorithms in terms of computational time is increased as per the suggestion of the respectable reviewer.

**Comment 3**: Real-world Scenario Testing for Latency and Network Overhead: "In the section on end-to-end latency and network overhead, it is suggested to add some test data from real network environments. These data can validate the performance of the proposed algorithm in real-world applications, especially in networks of different scales and densities."

**Answer:** We are thankful to the respectable reviewer for his kind suggestion. The proposed algorithm is simulated in a specialized MATLAB tool version 2021a through various realistic network scenarios with varying network density, transmission range and network area. We design multiple realistic scenarios with different network size (small, medium and large) i.e., 30-60 nodes in a network area (1-2 km) to validate the performance of the proposed algorithm. Various performance metrics such as latency and network overhead are recorded by varying transmission range in a simulation area of 1x1 km. The result demonstrate the efficacy of the proposed algorithms in terms of designated performance metrics.

**Comment 4**: Expansion of Future Work: "In the conclusion section, the directions for future work can be described more specifically, such as how to optimize the algorithm by integrating machine learning techniques, or how to further improve the algorithm to meet the specific needs of 5G and 6G networks. Detailed implementation plans and expected outcomes would help to enhance the feasibility of future work."

**Answer:** We are thankful to the respectable reviewer for his kind concern for improvement in the manuscript. The conclusion section is revised as per the direction of the respectable reviewer.

**Comment 5**: Explanation of Figures and Data: "The results presented in Figures 11, 12, and 13 should include more detailed explanations, such as the specific parameter settings of different algorithms and the network environment conditions. This information will help readers better understand the data and comparison results shown in the figures."

**Answer:** We are thankful to the respectable reviewer for his kind concern for improvement in the manuscript. More details regarding parameter settings and network environment conditions are included as per the direction of the respectable reviewer. Table 6 (simulation parameters) is also updated accordingly.

**Comment 6**: Writing and Formatting: “The overall writing of the article is smooth, but there are some minor errors, such as 'VAENT' should be 'VANET' and 'has' should be 'have.' It is recommended to carefully proofread the text. The format of the references needs to be consistent, and please ensure that all cited references are correctly listed."

**Answer:** We are thankful to the respectable reviewer for his kind concern for improvement in the manuscript. All the typo errors along with format of the references are corrected as per the recommendation of the respectable reviewer.

Reviewer 4:

**Comment 1**: In this work, the authors explore vehicular clustering as combinatorial optimization problem using the Arithmetic Optimization Algorithm (AOA) to address the issue of the 882 883 884 885 scalability and reliability of the vehicular network. This is a meaningful work. Following are some suggestions to the authors to improve the quality of the paper. The chapter numbering of the paper needs to be standardized. More details of the experimental environment should be explained. The abstract is too long. The author needs to add the latest literatures and analyze them, such as ppru: a privacy-preserving reputation updating scheme for cloud-assisted vehicular networks, trove: a context awareness trust model for VANETs using reinforcement learning. The captions of Figure5 to Figure13 should be rewritten. The conclusion is too long, please refine it. Please give specific strengths of the paper in the abstract and conclusion, expressed by specific numerical indicators.

Answer: We are thankful to the respectable reviewer for his kind concern for improvement in the manuscript. All the suggestions are taken accordingly and revised the manuscript as per the suggestion of the respectable reviewer. The abstract and conclusion is revised accordingly. Moreover, the references list is also corrected.

Versus
